# *CsPI* from the perianthless early-diverging *Chloranthus spicatus* show function on petal development in *Arabidopsis thaliana*

**DOI:** 10.1186/1999-3110-55-21

**Published:** 2014-02-04

**Authors:** Kunmei Su, Zhenhuan Li, Zhiduan Chen

**Affiliations:** 1 School of Environment and Chemistry Engineering, Tianjin Polytechnic University, Tianjin, 300387 China; 2 State Key Laboratory of Systematic and Evolutionary Botany, Institute of Botany, Chinese Academy of Sciences, Xiangshan, China

**Keywords:** Early-diverging angiosperm, B-class gene, *CsPI*, Perianthless, Petal development

## Abstract

**Background:**

In the floral ABC model, B-class genes comprised of *DEFICIENS* (*DEF*)/*APETALA3* (*AP3*) and *GLOBOSA* (*GLO*)/*PISTILLATA* (*PI*) had been proposed to involve in second and third whorl floral organ development. However, less is known about the function of B-class genes from early-diverging angiosperms. Chloranthaceae is one of the early-diverging angiosperm families. In this study, we characterized the role of the *PI*-like gene *CsPI* cloned from *Chloranthus spicatus* which have the simplest perianthless bisexual flowers.

**Results:**

The expression profile analysis reveals high levels of *CsPI* mRNA in stamens in *Chloranthus spicatus*, with weak distribution in leaves and other floral organs. Nevertheless, *CsPI* rescued both stamen and petal development in *Arabidopsis thaliana pi-1* mutants and caused partially conversion of sepals into petaloid organs in wild-type *Arabidopsis thaliana* plants. Yeast two-hybrid analysis showed that CsPI can form not only homodimers but also heterodimers with proteins encoded by *Arabidopsis thaliana* and *Chloranthus spicatus AP3*-like genes.

**Conclusions:**

These results suggested that *CsPI* has an ancestral function on stamen development and that *CsPI* has capability to specify petal development in *Arabidopsis thaliana.* The finding indicates that the activity of the encoded PI-like proteins is highly conserved between the early-diverging *Chloranthus* and *Arabidopsis*. Moreover, our results appear to suggest that B-function genes may not play a role in perianth development in *Chloranthus spicatus*.

**Electronic supplementary material:**

The online version of this article (doi:10.1186/1999-3110-55-21) contains supplementary material, which is available to authorized users.

## Background

In plants, MADS-box genes are of particular interest because of the large size of the family and the critical developmental roles the members are known to play (Theissen et al. 2000). In the model plant *Arabidopsis thaliana*, five classes of MADS-box genes were involved in determing the development of floral organ identity. Functions of these genes have been summarized in the ABCDE model, which holds that different A, B, C, D and E class MADS-domain proteins interact to form functional “ternary” or “quartet” protein complexes that are responsible for establishing the various floral organ identities (Egea-Cortines et al. 1999; Honma and Goto 2001; Smaczniaka et al. 2012; Theissen and Saedler 2001). In this model, the A class genes *APETALA1* (*AP1*) and *APETALA2* (*AP2*) control sepal formation; A, B [*APETALA3* (*AP3*), *PISTILLATA* (*PI*)] and E (*SEPALLATA1*/*2*/*3*) class genes together regulate petal formation; B, C [*AGAMOUS* (*AG*)] and E class genes control stamen formation; C and E class genes regulate carpel formation; and the D class genes *SEEDSTICK* (*STK*) are involved in ovule development (Theissen 2001; Theissen and Saedler 2001).

Numbers of MADS-box genes have already been identified in almost every group of flowering plants, including early-diverging angiosperms. These MADS-box genes involved in flower development provided convenience for further studies on the evolution of flowers. Up to now, a huge variety of inflorescence and floral morphologies are found among flowering plants. Phylogenetic studies based on morphology and genes have demonstrated that the origin and early diversification of flowers during evolution may have significantly contributed to the sudden occurrence of diverse angiosperms in a relatively short time span during the Early Cretaceous. Therefore, the MADS-box gene family controlling flower development in early-diverging plants gains more and more attention.

Among the early-diverging angiosperms, the ANITA groups (ANITA is the acronym of Amborella, Nymphaeaceae, Illiciales, Trimeniaceae and Austrobaileyaceae), which have undifferentiated perianth, are suggested the earliest extant angiosperms by phylogeny analysis (Hansen et al. 2007; Soltis et al. 2007a; Qiu et al. 1999; Zanis et al. 2002). Following this earliest diverging grade, Chloranthaceae is sister to the magnoliids and together this group is sister to a large clade that includes eudicots and monocots (Hansen et al. 2007; Moore et al. 2007). In fact, the family Chloranthaceae has been placed in many different positions in phylogenetic trees based on morphology and gene sequences, for example Piperales, Laurales, Magnoliales, Austrobaileyales (reviewed by Hansen et al. 2007). The family Chloranthaceae contains four extant genera (Chloranthus, Sarcandra, Ascarina, and Hedyosmum) and approximately 70 species. Each of the four extant genera has distinctive morphological: *Chloranthus* and *Sarcandra* possess the simplest bisexual flowers in angiosperms; *Ascrina* and *Hedyosmum*, however, bear the simplest unisexual flowers in angiosperms. Thus Chloranthaceae represents an interesting model with which to explore the evolution of flowers.

In the floral ABC model, B-class genes comprised of *DEFICIENS* (*DEF*)/*APETALA3* (*AP3*) and *GLOBOSA* (*GLO*)/*PISTILLATA* (*PI*) had been proposed to involve in second and third whorl organ development. In eudicots, functions of *AP3-* like and *PI-* like genes are basically conserved in petal and stamen development (for review Soltis et al. 2007b; Becker and Theissen 2003). In the core eudicot *A. thaliana*, single mutant of *AP3* and *PI* caused the homeotic transformation of petals to sepals in the second whorl and of stamens to carpels in the third whorl (Jack et al. 1992; Goto and Meyerowitz 1994). In basal eudicots *Aquilegia vulgaris* and *Papaver somniferum* (Ranunculales), B-class genes are also found to be necessary for the development of both petals and stamens (Drea et al. 2007; Kramer et al. 2007). In the basal eudicot California poppy (*Eschscholzia californica*), mutant of the *PI-* lineage gene *SEI* shows homeotic changes characteristic of floral homeotic B class mutants (Lange et al. 2013). In monocots, heterologous expression studies suggested that B-class genes play the same role as in eudicots, although data from heterologous expression studies are difficult to interpret (Bartlett and Specht 2010). *silky1* (*si1*), a mutant of Zea mays *AP3*-like gene, shows homeotic conversions of stamens into carpels and lodicules into palea/lemma-like structures (Ambrose et al. 2000). Consistent with this, *Silky1* and *Zmm16* (*PI*-like gene of Zea mays), are also able to rescue petal development in *A. thaliana ap3* and *pi* mutant, respectively (Whipple et al. 2004). The *PI* homologs from *Agapanthus praecox* and *Elaeis guineensis*, monocot flowers with petaloid inner perianth organs, also have been shown to rescue the *pi-1* mutant of *A. thaliana* (Nakamura et al. 2005; Adam et al. 2007). These data appear to suggest that the function of B-class genes is conserved in monocots and eudicots. However, less is known about the function of B-class genes in early-diverging angiosperms. Therefore, we preferentially selected the B class genes from the early-diverging angiosperm *Chlornthus spicatus* for functional analysis.

In *Chlornthus spicatus*, the *AP3*-like gene *CsAP3* have been investigated through in situ hybridization expression analyses and transformation experiments. *CsAP3* is exclusively expressed in male floral organs, but is not detected in the dome-shaped spike primordia, bract primordial and leaves (Li et al. 2005). Only weak complementation was seen in the third floral whorl (stamen), nevertheless, no complementation was seen in the second floral whorl (petal) when *CsAP3* was expressed in *A. thaliana ap3-3* mutant plants (Su et al. 2008). No ectopic gain-of-function in the fourth floral whorl was observed when *CsAP3* was ectopically expressed in wild-type *A. thaliana* plants. However, less research work on the function of the *PI*-like gene from *C. spicatus* was reported although complete coding sequence of *CsPI* has already been isolated previously (Su et al. 2008). Therefore, functional analysis of *CsPI* is necessary.

To investigate the role of the *PI*-like gene *CsPI* in floral development, the expression pattern was analyzed using quantitative real-time PCR analysis. To complement the results of the expression pattern analyses, we transformed *35S*::*CsPI* into wild-type *A. thaliana* plants and *5D3*::*CsPI* into the *pi-1* mutant plants. To explore how they worked, we tested interactions of proteins by employing the yeast two-hybrid system.

## Methods

### Plant material and RNA extraction

*C. spicatus* used in our experiments were cultivated in the Botanical Garden, Institute of Botany, Chinese Academy of Sciences, Beijing. Total RNA was prepared using Trizol (Invitrogen). Then poly(A) mRNA were purified using Oligotex mRNA Mini Kit (Qiagen) and the first-strand cDNA was synthesized with Superscript III (Invitrogen) (Su et al. 2008).

### Vectors construction

Full-length *CsPI* cDNA sequence fragment was cloned into the binary vector pCAMBIA 1301 (Cpgbiotech). Primers YCsPI and PTA were used in PCR amplification. The cauliflower mosaic virus (CaMV) 35S promoter (Benfey and Chua 1990) was fused to the cDNA to drive nearly ubiquitous expression of all the transgenes in a wild-type background. Furthermore, to avoid ectopic expression of these transgenes, in another series of experiments the *A. thaliana AP3* promoter *5D3* was used to drive expression of the transgenes in whorls 2 and 3 of developing *A. thaliana* flowers in the *pi-1* mutant background (Lamb and Irish 2003). The promoter sequence was amplified by PCR from DNA extracted from leaves of wild-type *A. thaliana* using primers in our previous studies (Su et al. 2008).

### *A. thaliana* transformation and genotyping

The plasmid constructs were transformed into wild-type *Landsberg erecta A. thaliana* plants and *pi-1* mutant plants respectively, by the floral dip method (Clough and Bent 1998).

Seeds of the transgenic *A. thaliana* plants were selected on solid 0.5 × MS medium (Murashige and Skoog 1962) containing 50 mg/L rifampicin at 4°C for 2 days, and then were transferred to the greenhouse under long-day condition (16 h light/8 h dark) at 22°C for 10 days. As the control, seeds of wild-type *A. thaliana* were cultured on solid 0.5 × MS medium as described above. Subsequently, the wild-type and transgenic seedlings were transplanted to soil and were cultured at 22°C with 16 h light and 8 h dark.

Homozygous *pi-1* plants were identified using a dCAPS marker, in which BspHI cuts the wild-type sequence (Lamb and Irish 2003), but the site is abolished by the *pi-1* mutation. All observed phenotypes were heritable and segregated as dominant traits. Morphological analysis was performed on the T1 generation.

### Primers used in experiments

Primers used in our experiments were all showed in Table 1.

**Table 1:** **Primers used in this paper**

Name of primers	Sequence of primers
CsPIReTi-F2	5′-GCGTTTAAGCTACATCTTGCATC-3’
CsPIRETI-R2	5′-ATGGTTCTGGTGGAAACGAAG-3’
qActup	5′-CGTATGAGCAAGGAGATCAC-3’
qActdown	5′-CACATCTGTTGGAAGGTGCT-3’
18S primerF	5′-CGGCTACCACATCCAAGGAA-3’
18S primerR	5′-TGTCACTACCTCCCCGTGTCA-3’
AtPINde1	5′-GATCTCATATGGGTAGAGGAAAG-3’
AtPINoM	5′-TGATTGAATTCTGTTGTCCTTCCATG-3’
YCsAP3	5′-CGGGCCATGGGAAGAGGAAAGATT-3’
CsAP3NoM	5′-TCTATCATATGTGCAGCCCTGCTAC-3’
YCsPI	5′-CGGGCCATGGGTCGTGGGAAGATC-3’
CsPINoM	5′-TGTTCGAATTCGTTAGCCCCTCTAC-3’
AtAP3Nde1	5′-GATCTCATATGGCGAGAGGGAAG-3’
AtAP3NoM	5′-TTCATGAATTCATCAGCCCTAACAC-3’
PIINT-2	5′-CCAATTTCATGATATCTAGCTCAG-3’
PI-1	5′-TACCAGAAGTTATCTGGCAAGAAATCATCATG-3’
PTA	5′-CCGGATCCTCTAGAGCGGCCGC(T)_17_-3’

### Quantitative real-time PCR analysis

Total RNA was extracted from roots, stems, leaves, bracts, stamens and carpels of *C. spicatus* for expression pattern analysis of *CsPI*. For their constitutive and complementary expression analysis, total RNA was extracted from the inflorescences of *A. thaliana* carrying transgenic constructs. After the purification of RNA samples, first-strand cDNA was synthesized with Superscript™ III Reverse Transcriptase (Invitrogen) in a 20μl reaction volume. Each kind of sample was prepared three times as described above. Quantitative real-time PCR was performed with the iQ SYBR Green supermix (Bio-Rad) in a Rotor-gene 3000 classic real-time PCR machine (Corbett Research). PCR conditions were 15 min at 95°C, followed by 40 cycles of 30 s at 94°C, 30 s at 56°C and 30 s at 72°C. To detect the expression pattern of *CsPI* in *C. spicatus*, the *C. spicatus* housekeeping gene 18S rRNA was used to normalize the amount of the cDNAs added to the reaction. To analysis the expression of *CsPI* in wild-type and *pi-1* mutant *A. thaliana*, the *A. thaliana* housekeeping gene *ACTIN* was used as normalization control. Specific primer pairs were designed with the help of Beacon Designer 4 software (Premier Biosoft International). These primers include CsPIReTi-F2, CsPIRETI-R2. In each experiment, two standard curves were applied for the relative quantification of the cDNA copies. Each sample was analyzed three times to determine reproducibility.

### SEM observation

All flowers collected from the transgenic wild-type *A. thaliana* plants were immediately fixed with FAA (formalin: acetic acid: 50% ethanol = 5: 6: 89). Then these flowers were dried and coated as described previously (Xu et al. 2005), and observed with a Hitachi S-800 scanning electron microscope (SEM).

### Yeast two-hybrid assays

Yeast two-hybrid assays were performed using the GAL4-based MATCHMAKER Two-Hybrid System (Clontech). *Saccharomyces cerevisiae* strain AH109, GAL4 activation domain (AD) expression vector pGADT7 and GAL4 DNA-binding domain (DNA-BD) expression vector pGBKT7 were used. Full-length cDNA of *CsAP3*, *CsPI*, were amplified with *Nco* I restriction enzymes digest site overlapping the start codon and *Bam* HI at the 3′ end. *Eco* RI and *Bam* HI sites were introduced to generate MADS-deleted *CsAP3* and *CsPI*, for cloning into pGADT7 and pGBKT7, respectively. All constructs were verified by restriction enzymes analyses and sequencing. The yeast strain AH109 was transformed with above constructs according to the manufacture’s protocol of small-scale LiAc yeast transformation procedure. Confirmation of the transformants and interaction analyses were performed as previously described (Shan et al. 2006; Su et al. 2008). The transformants co-transformed plasmids of AP3 and PI in absence of MADS domain from *A. thaliana* were used as a positive control (Yang et al. 2003). The transformants containing plasmids pGADT7 and pGBKT7 were used as a negative control.

## Results

### Expression patterns of *CsPI* in *C. spicatus*

**Figure 1:**
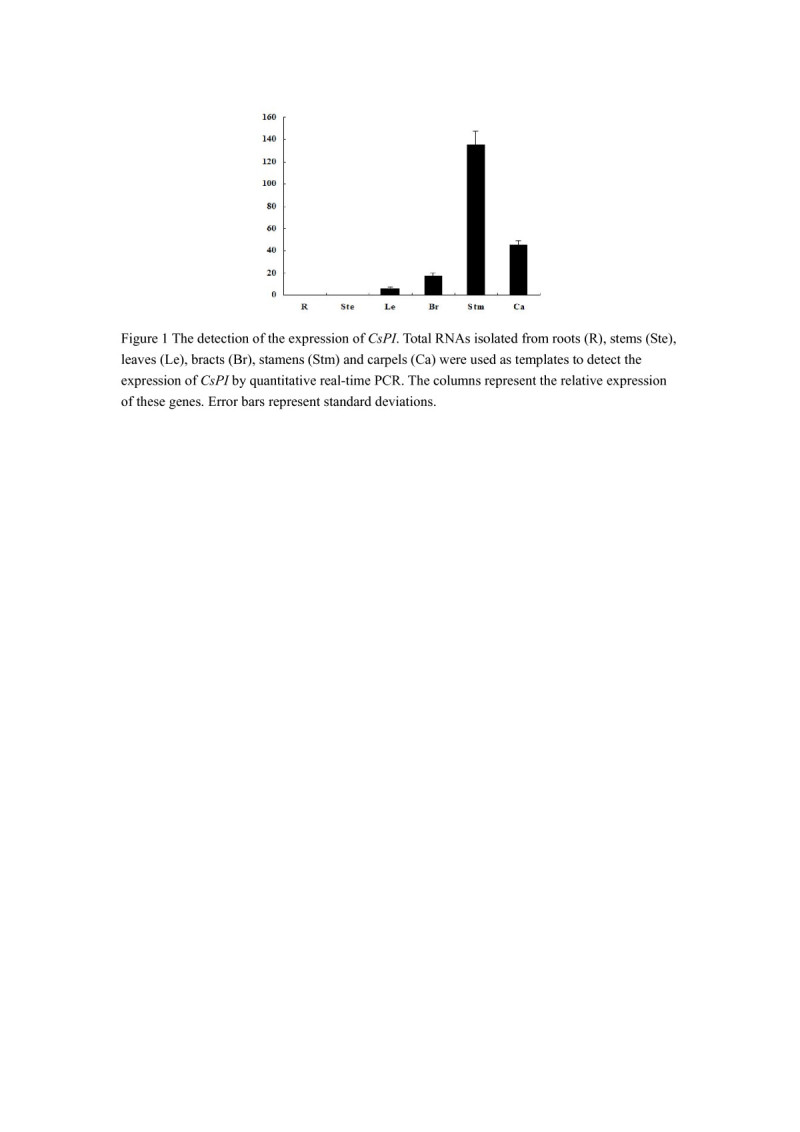
**The detection of the expression of** ***CsPI*** **.** Total RNAs isolated from roots (R), stems (Ste), leaves (Le), bracts (Br), stamens (Stm) and carpels (Ca) were used as templates to detect the expression of *CsPI* by quantitative real-time PCR. The columns represent the relative expression of these genes. Error bars represent standard deviations.

In order to get a clue about the function of *CsPI*, mRNA accumulation was analyzed by quantitative real-time PCR. As shown in Figure 1, *CsPI* mRNA was absent in roots and stems. Only weak expression of *CsPI* was found in leaves and bracts (Figure 1). Some expression was expressed in carpels and the strongest expression was detected in stamens (Figure 1). The expressing quantity of *CsPI* in stamens was 3 times what in carpels. These data suggested that *CsPI* was expressed broadly in *C. spicatus*. The expression pattern is similar to those of the *PI*-like genes from other early-diverging angiosperms (Kim et al. 2005; Lv et al. 2012).

### Ectopic expression of *CsPI* in wild-type *A. thaliana*

To further explore the function of *CsPI* in floral development, we transformed wild-type *A. thaliana* plants with the cDNA under the control of the cauliflower mosaic virus (CaMV) 35S promoter.

**Figure 2:**
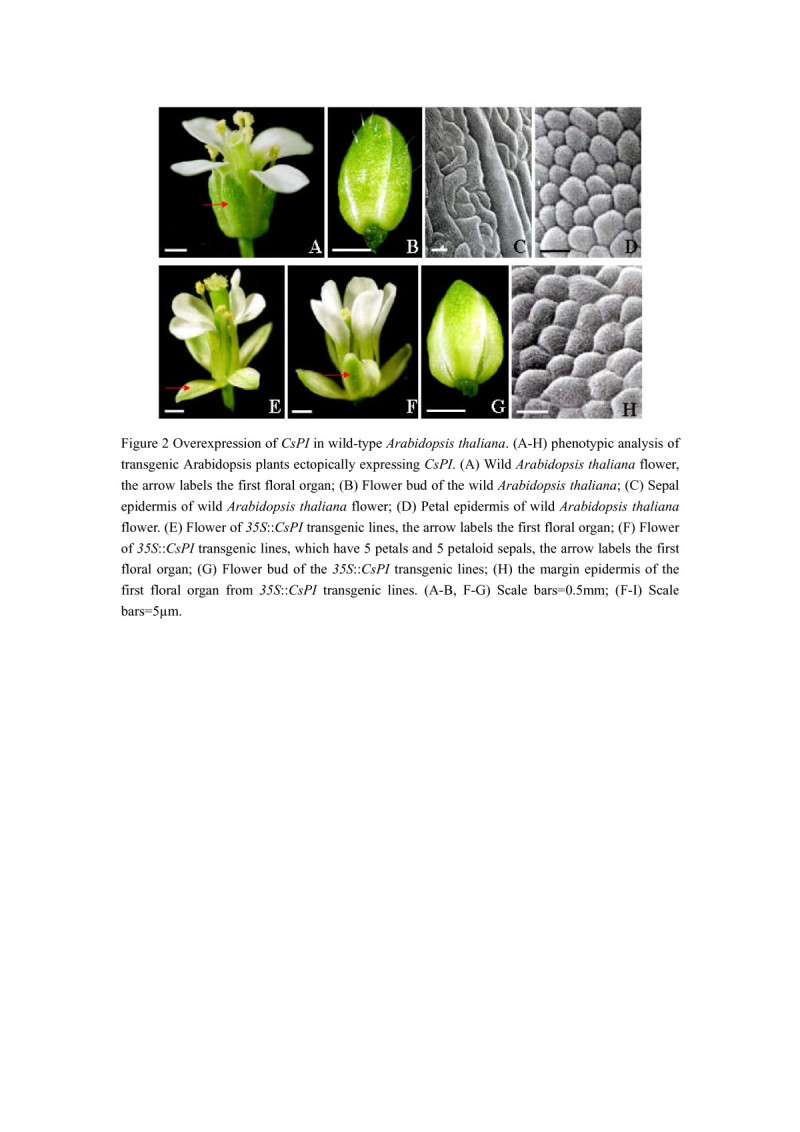
**Overexpression of** ***CsPI*** **in wild-type** ***Arabidopsis thaliana*** **. (A-H)** phenotypic analysis of transgenic *Arabidopsis thaliana* plants ectopically expressing *CsPI*. **(A)** Wild *Arabidopsis thaliana* flower, the arrow labels the first floral organ; **(B)** Flower bud of the wild *Arabidopsis thaliana*; **(C)** Sepal epidermis of wild *Arabidopsis thaliana* flower; **(D)** Petal epidermis of wild *Arabidopsis thaliana* flower. **(E)** Flower of *35S*::*CsPI* transgenic lines, the arrow labels the first floral organ; **(F)** Flower of *35S*::*CsPI* transgenic lines, which have 5 petals and 5 petaloid sepals, the arrow labels the first floral organ; **(G)** Flower bud of the *35S*::*CsPI* transgenic lines; **(H)** the margin epidermis of the first floral organ from *35S*::*CsPI* transgenic lines. **(A-B, F-G)** Scale bars = 0.5 mm; **(F-I)** Scale bars = 5 μm.

**Figure 3:**
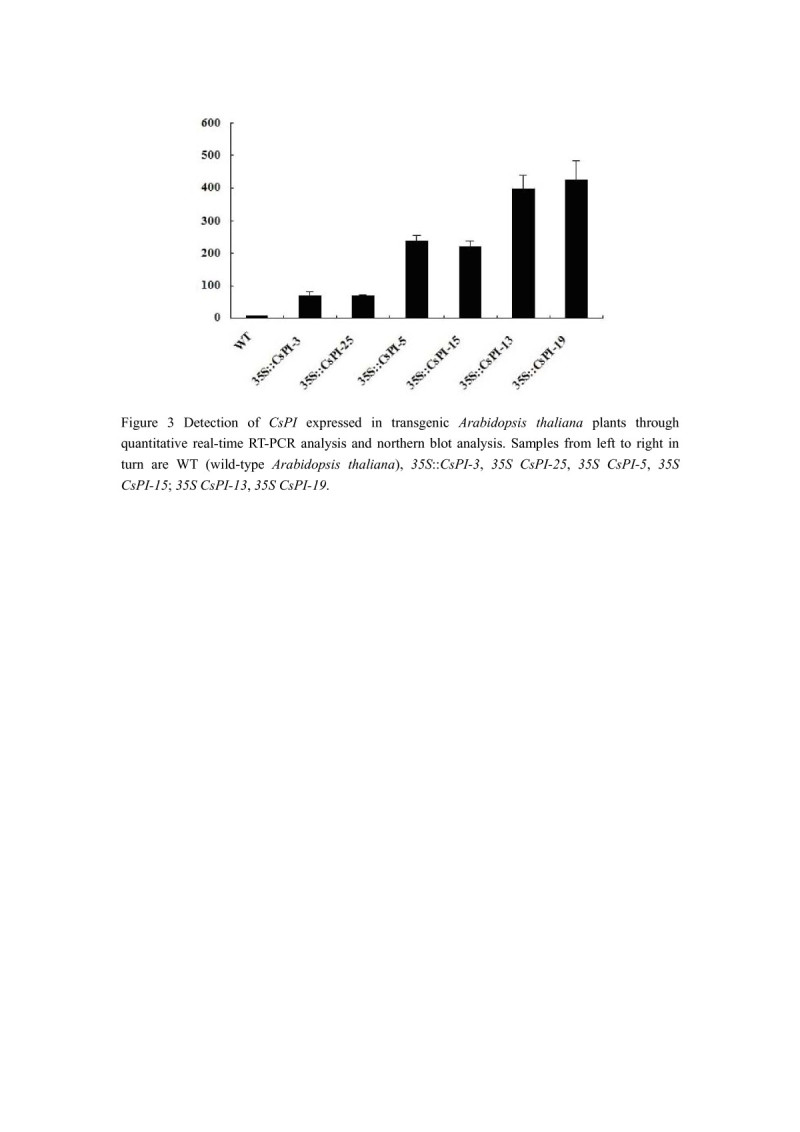
**Detection of** ***CsPI*** **expressed in transgenic** ***Arabidopsis thaliana*** **plants through quantitative real-time PCR analysis and northern blot analysis.** Samples from left to right in turn are WT (wild-type *Arabidopsis thaliana*), *35S*::*CsPI-3*, *35S CsPI-25*, *35S CsPI-5*, *35S CsPI-15*; *35S CsPI-13*, *35S CsPI-19*.

We obtained 42 *A. thaliana* transgenic plants, 26 of which displayed homeotic changes. The vegetative organs of these plants were normal, and no effect in flowering time was detected (data not shown). Phenotypic alterations were observed only in flowers. Flowers of these *35S*::*CsPI* transgenic plants seemed to have two whorls of petals (Figure 2E and F). Sepals in the first whorl were partially converted into petaloid organs (Figure 2E, F, G). These petaloid structures expanded like petals although its size was smaller than that of petals (Figure 3E and F). Moreover, flowers of some *35S*::*CsPI* transgenic plants, such as line *13* and *19* showed 5 petals and 5 petaloid sepals (Figure 2F). Noticeably different from those of the wild-type flowers, margins of these petaloid sepals consist of white tissue and surface were smooth (Figure 2F and G, compare F with A and G with B separately). Examination by SEM revealed that the surface of these regions in the *35S*::*CsPI* transgenic plants was a mosaic composed of both sepal and petal cells, while these cells were similar in shape and size (Figure 2H, compare H with C, D). However, flowers of *35S*::*CsPI-3 and 35S*::*CsPI-25 were similar to wild-type A. thaliana*. To find whether the severe phenotypes were correlated with *CsPI* expression in the transgenic plants, quantitative real-time PCR analysis was performed. Transgenic lines *with only* 4 petals and 4 petaloid sepals, represented by *35S*::*CsPI-5 and 35S*::*CsPI-15,* showed lesser RNA expression of *CsPI than 35S*::*CsPI-13 and 35S*::*CsPI-19* (Figure 3). However, the expression of *CsPI* in these 4 lines was obviously higher than what in lines *35S*::*CsPI-3 and 35S*::*CsPI-25.* These data demonstrated that the accumulation levels of *CsPI* transcripts in different lines are consistent with phenotypic alterations.

### Functionality of *CsPI* in *pi-1* mutants of *A. thaliana*

In addition to the wild-type *A. thaliana*, *CsPI* was also transformed into *A. thaliana pi-1* mutant plants. In this transformation experiments, the *A. thaliana AP3* promoter *5D3* was used to drive expression of *CsPI* in whorls 2 and 3 of developing *A. thaliana* flowers in the *pi-1* mutant background (Lamb and Irish 2003).

**Figure 4:**
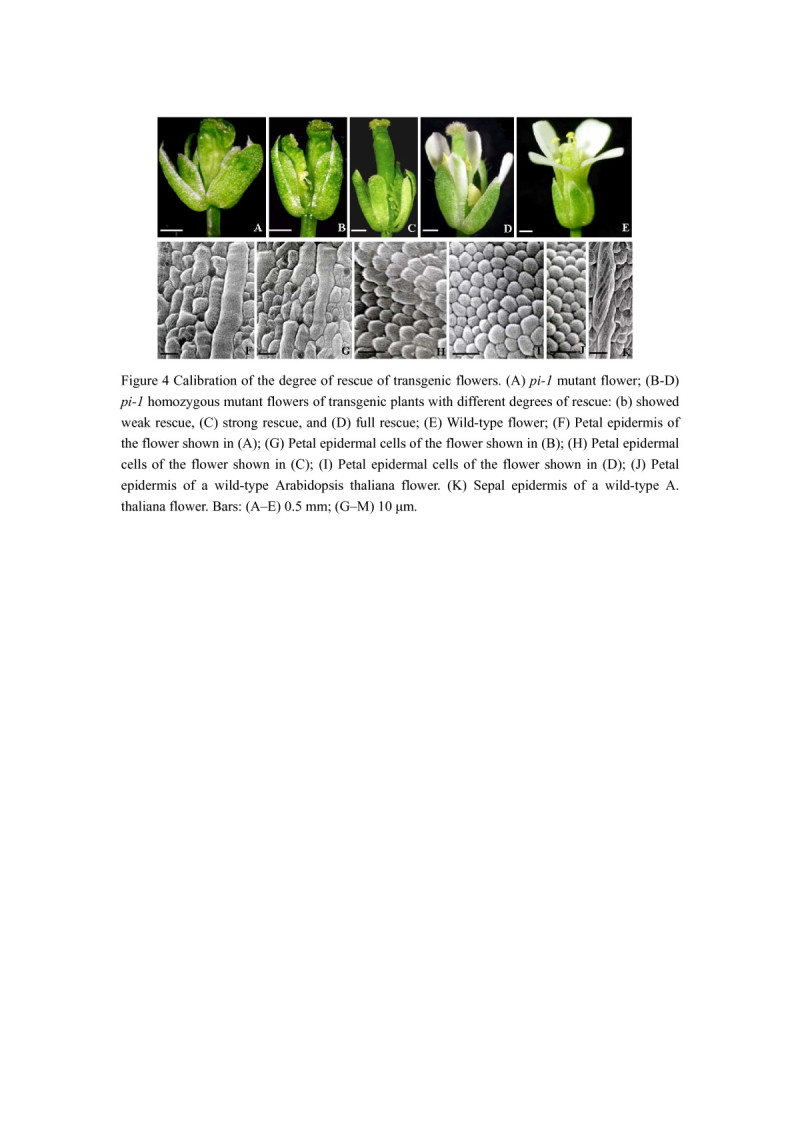
**Calibration of the degree of rescue of transgenic flowers. (A)** *pi-1* mutant flower; **(B-D)** *pi-1* homozygous mutant flowers of transgenic plants with different degrees of rescue: **(B)** showed weak rescue, the arrow labels the mosaic stamen, **(C)** strong rescue, and **(D)** full rescue; **(E)** Wild-type flower; **(F)** Petal epidermis of the flower shown in **(A)**; (G) Petal epidermal cells of the flower shown in **(B)**; **(H)** Petal epidermal cells of the flower shown in **(C)**; **(I)** Petal epidermal cells of the flower shown in **(D)**; **(J)** Petal epidermis of a wild-type *Arabidopsis thaliana* flower. **(K)** Sepal epidermis of a wild-type A. thaliana flower. Bars: **(A–E)** 0.5 mm; (G–M) 10 μm.

We obtained 21 independent *5D3*::*CsPI* transgenic *pi-1* plants. Among of them, 10 (47.6%) showed full rescue and 4 (19%) showed strong rescue. In flowers of fully rescued plants, petals had the shape of wild-type petals but were somewhat smaller (Figure 4D). Moreover, the epidermal cells of rescued petals (Figure 4I) resembled those of the wild-type which were characteristically rounded (Figure 4J). Petals of strongly rescued flowers were small and green (Figure 4C), with the epidermal petal cells which were more similar to those of wild-type petals than sepals (Figure 4H). The third-whorl floral organs of fully rescued flowers were not fully extended stamens with fertile pollen grains (Figure 4D), while the third floral whorl of strongly rescued flowers were mosaic organs between carpel and stamen (Figure 4E). Weak rescue was also seen for 7 (33.3%) lines, in which neither stamens nor petals were rescued (Figure 4B).

**Figure 5:**
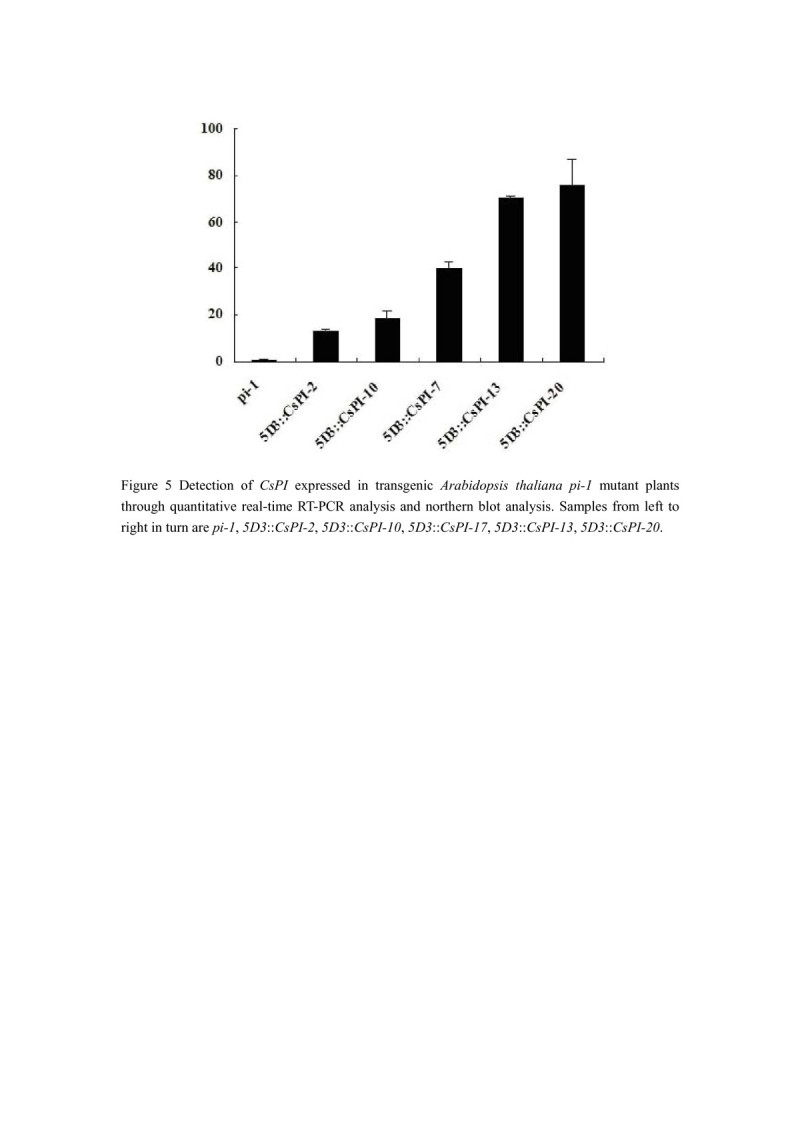
**Detection of** ***CsPI*** **expressed in transgenic** ***Arabidopsis thaliana pi-1*** **mutant plants through quantitative real-time PCR analysis and northern blot analysis.** Samples from left to right in turn are *pi-1*, *5D3*::*CsPI-2*, *5D3*::*CsPI-10*, *5D3*::*CsPI-17*, *5D3*::*CsPI-13*, *5D3*::*CsPI-20*.

Here, transgene expression was also determined by quantitative real-time PCR, which demonstrated that level of phenotypic rescue is correlated with the expression level of transgene (Figure 5). For example, the expression of *CsPI* was clearly higher in fully rescued *5D3*::*CsPI-* 13 and *5D3*::*CsPI-* 20 than in strongly rescued *5D3*::*CsPI-* 7, while the expression of *CsPI* was clearly lesser in weakly rescued *5D3*::*CsPI*-2 and *5D3*::*CsPI*-5 than in strongly rescued *5D3*::*CsPI-* 7.

### Interaction pattern analysis of CsPI

To investigate the interaction patterns of CsPI proteins to learn how they worked, yeast two-hybrid assays were performed. As positive control, we investigated the interaction between *A. thaliana* AP3 and PI proteins, which was marked as AtAP3 and AtPI respectively. As negative controls, we detected the growth of transformants co-transformed with the fusion plasmid containing the protein and the pGADT7 or the pGBKT7 free vector.

**Figure 6:**
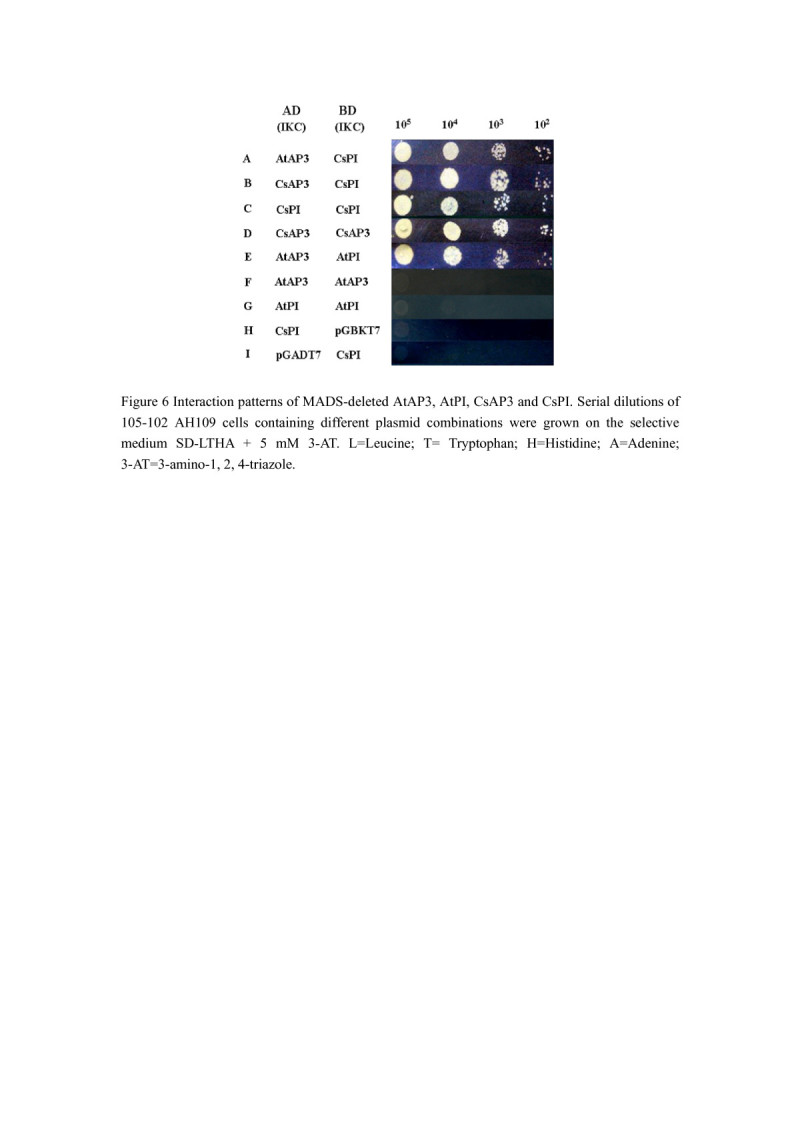
**Interaction patterns of MADS-deleted AtAP3, AtPI, CsAP3 and CsPI.** Serial dilutions of 105–102 AH109 cells containing different plasmid combinations were grown on the selective medium SD-LTHA + 5 mM 3-AT. L = Leucine; T = Tryptophan; H = Histidine; A = Adenine; 3-AT = 3-amino-1, 2, 4-triazole.

In our experiments, interaction patterns of the full-length and the MADS-deleted CsPI, CsAP3, AtPI and AtAP3 were tested. As negative controls, we demonstrated that transformants co-transformed with the fusion plasmid containing the protein and the pGADT7 or the pGBKT7 free vector did not grow on the selective medium (Figure 6H, I). As positive control, the MADS-deleted AtPI and AtAP3 sequence formed heterodimers (Figure 6) (Yang et al. 2003). Dimerization could not be observed for full-length CsPI, CsAP3, AtPI and AtAP3 (data not shown). However, the MADS-deleted CsPI can form heterodimers with AtAP3 and CsAP3 (Figure 6 A and B). Since specificity of heterodimerization is largely based on the sequence of the I-domain and K-domain (Kaufmann et al. 2005; Riechmann et al. 1996; Yang et al. 2003), this applies very likely also to the full length (MIKC) sequence. Moreover, the MADS-deleted CsPI can also form homodimerization (Figure 6C), a feature which has been found also for some other AP3-like and PI-like proteins of non-core eudicots, including monocots such as lily (*Lilium*) and tulip (*Tulipa*), but not in core eudicots (Hsu and Yang 2002; Su et al. 2008; Tzeng et al. 2004; Winter et al. 2002). However, the MADS-deleted protein AtPI was not able to interact with itself (Figure 6G).

## Discussion

According to the ABCDE model, B class genes, including both *PISTILLATA* (*PI*) and *APETALA3* (*AP3*) homologs, contribute to petal and stamen development. Functional analysis concentrated on monocots and eudicots suggested that the function of the B-class genes is conserved. In this study, we demonstrated the functional conservation of the *PI*-like genes between the early-diverging angiosperm *C. spicatus* and *A. thaliana*.

To identify the function of *CsPI* in *C. spicatus*, we detected the expression pattern through quantitative real-time PCR. *CsPI* was expressed in a broad range, including the leaves, bracts, stamens and carpels. The expression pattern of *CsPI* was different from that of the other *C. spicatus* B-class gene *CsAP3*, which was found to be exclusively expressed in stamens (Li et al. 2005). The expression pattern was also different with that of the core eudicots *AP3*/*PI* genes, which are expressed restricted to the second and third whorls (reviewed by Kim et al. 2005). However, this pattern was consistent with those of their counterparts in early-diverging angiosperms. Kim et al. also found that PI transcripts were detected in petals, stamens and carpels in early-diverging such as in *Amborella trichopoda* and *Nuphar advena* (Kim et al. 2005). Similarly, *AcPI* in monocot *Ananas comosus* was expressed in stems, leaves, bracts and sepals, petals, stamens and carpels (Lv et al. 2012). The broader range of expression of *PI* homologs is inferred to be the ancestral pattern for all angiosperms (Kim et al. 2005). However, it is worth mentioning that strong expression of *CsPI* was only detected in stamens. Although MADS-box gene function is often correlated with gene expression pattern, transient and/or weak expression does not correspond to a known genetic function (reviewed by Kim et al. 2005). Therefore, *CsPI* may mainly function on stamen development in *C. spicatus*. Compatible with this hypothesis, the complementation of the third whorl floral organs of the *pi-1* mutant plants were observed when *CsPI* was expressed under the control of the *AP3* promoter *5D3*. The phenotype is also observed in *pi* mutant plants transformed with *PI* and the *PI*-like gene *Zmm16* from maize under the control of the *A. thaliana AP3* promoter (Lamb and Irish 2003; Piwarzyk et al. 2007; Whipple et al. 2004). These results suggested that the *PI*-like gene *CsPI* from the early-diverging *C. spicatus* conserved the function on stamen development.

Most interestingly, *CsPI* also showed function on the petal development when it was expressed in wild-type or *pi* mutant *A. thaliana* plants. Like to those of the *A. thaliana pi* mutant plants expressing *PI* or the *PI*-like gene *Zmm16* (Lamb and Irish 2003; Piwarzyk et al. 2007; Whipple et al. 2004; Yang et al. 2003), the second whorl floral organs were rescued when *5D3*::*CsPI* was transformed into *pi-1* mutant plants. In line with this, the *35S::CsPI* transgenic plants exhibited a partial conversion of sepals to petaloid organs. This phenotype is similar to that of the *35S*::*PI A. thaliana* plants. It has been reported that the *35S*::*PI A. thaliana* also modifies sepals into petaloid organs but no ectopic stamen is formed (Krizek and Meyerowitz 1996; Lamb and Irish 2003; Yang et al. 2003). The only slight difference is that flowers of some *35S*::*CsPI* plants showed an increase in the number of the first and the second floral organs. This can be attributed to the different expression levels as shown in quantitative real-time PCR analysis and northern blot analysis. Alternatively, the expression level of *CsPI* may be correlated with the number of petals.

As to why *CsPI* showed functions in *A. thaliana* similar to those of *PI*, it is possibly provided by the yeast two-hybrid analysis, which revealed that CsPI proteins can form heterodimers with AtAP3 proteins. It has been reported that the *A. thaliana AtAP3* gene was faintly expressed in the first floral organ as well as in the second and the third floral organs (Jack et al. 1992; Smaczniaka et al. 2012). Therefore, the fact of transforming sepal into petalloid structures or rescue the second and the third whorl of the *pi-1* mutant plants might be due to the same fact as that of the *A. thaliana* genes, both *AP3* and *PI* should be present together with *SEP* genes (Krizek and Meyerowitz 1996).

Alternatively, homodimers of CsPI may also be able to act to specify petals with AtAP3. As shown, CsPI can form homodimers besides heterodimers. This feature also has been found for some other class B proteins of gymnosperms and non-core eudicots (Chen et al. 2012; Hsu and Yang 2002; Liu et al. 2013; Liu et al. 2010; Su et al. 2008; Tzeng et al. 2004; Winter et al. 2002; Yang et al. 2003), but not in core eudicots. For example, proteins transformed by *Lilium longiflorum PI*-like genes *LMADS8* and *LMADS9* can also form homodimers besides heterodimers (Chen et al. 2012). It’s worth noting that flowers of the *A. thaliana* overexpressed the *Lilium longiflorum LMADS8/9* (*PI*-like) also showed partially transformation of sepals to petaloid organs and homodimers of *LMADS8/9* were able to bind to the CArG1 of AtAP3 (Chen et al. 2012). Moreover, C-terminal deleted HoPI (PI-like) proteins from *Hedyosmum orientale* (Chloranthaceae) lost the petal identity function in *A. thaliana* as they failed to form homodimers (Liu et al. 2013). For these facts, we can’t exclude such a scenario for homodimers of CsPI to act in petal development in *A. thaliana*. This interaction pattern may represent an ancient flexible interaction of AP3 and PI lineage proteins (Liu et al. 2013).

This finding that *CsPI* has capability to specify petal identity in *A. thaliana* was compatible with the view that the perianthless state of *C. spicatus* is derived rather than ancestral (Li et al. 2005). As to the loss of petals, we prefer the hypothesis that the B function,which requires the concerted expression of AP3 and PI homologues, may not contribute to petal development in Chloranthaceae (Liu et al. 2013). In *H. orientale* (Chloranthaceae), *HoPI* was broadly expressed in all floral organs, whereas *HoAP3* was restricted to stamens (Liu et al. 2013). In perianthless *C. spicatus*, *CsPI* reported here, was also broadly expressed in all floral organs, but *CsAP3* was restricted to stamens (Li et al. 2005). Therefore, the overlap of *AP3* and *PI* homologue expression is limited to the stamens in Chloranthaceae. Yet, coordinated expression of the *AP3-* and *PI-* like genes is correlated with the identity of petaloid organs (reviewed by Liu et al. 2013). These data appear to suggest that the main reason for the loss of petals in Chloranthaceae maybe not the floral homeotic B-function. Nonetheless, we still can’t rule out the possibility that changes in cis-regulatory elements or trans-regulatory factors that regulate B-class genes are causally linked to the greatly reduced perianth in *Chloranthus* (Li et al. 2005). As shown in this paper, some *35S*::*CsPI* plants showed an increase in the number of the first and the second floral organs. These plants showed expression of *CsPI* which was much higher than that of other plants. The data implied that weak expression of B class genes in *C. spicatus* may be correlated with the reduction of perianth. Consistent with this hypothesis, it has been reported that independent petal losses within buttercup family (Ranunculaceae) were strongly associated with decreased or eliminated expression of a B-class gene, *APETALA3-3 (AP3-3)* (Zhang et al. 2013). It would be interesting to investigate, therefore, whether there are specific cis-regulatory elements controlling the expression of *CsAP3* and *CsPI* in petals.

## Conclusions

*CsPI* retained the ancestral function in stamen identity and showed capability to specify petal development in *A. thaliana*. These data suggested that the role of *PI*-like gene was conserved in the early-diverging angiosperm *Chloranthus spicatus* and the core-eudicot *Arabidopsis thaliana*. CsPI can form homodimers besides heterodimers and they may both be involved in petal development in *A. thaliana*. Moreover, it seems likely that the loss of petals maybe not directly caused by the floral homeotic B-function in *Chloranthus spicatus*.

## Authors’ original submitted files for images

Below are the links to the authors’ original submitted files for images. Authors’ original file for figure 1 Authors’ original file for figure 2 Authors’ original file for figure 3 Authors’ original file for figure 4 Authors’ original file for figure 5 Authors’ original file for figure 6
