# Supplementary figures and images for: CsPI from the perianthless early-diverging Chloranthus spicatus show function on petal development in Arabidopsis thaliana
Source: Bot Stud. 2014 Feb 4;55:21. doi: 10.1186/1999-3110-55-21 (PMC5430364; doi:10.1186/1999-3110-55-21)

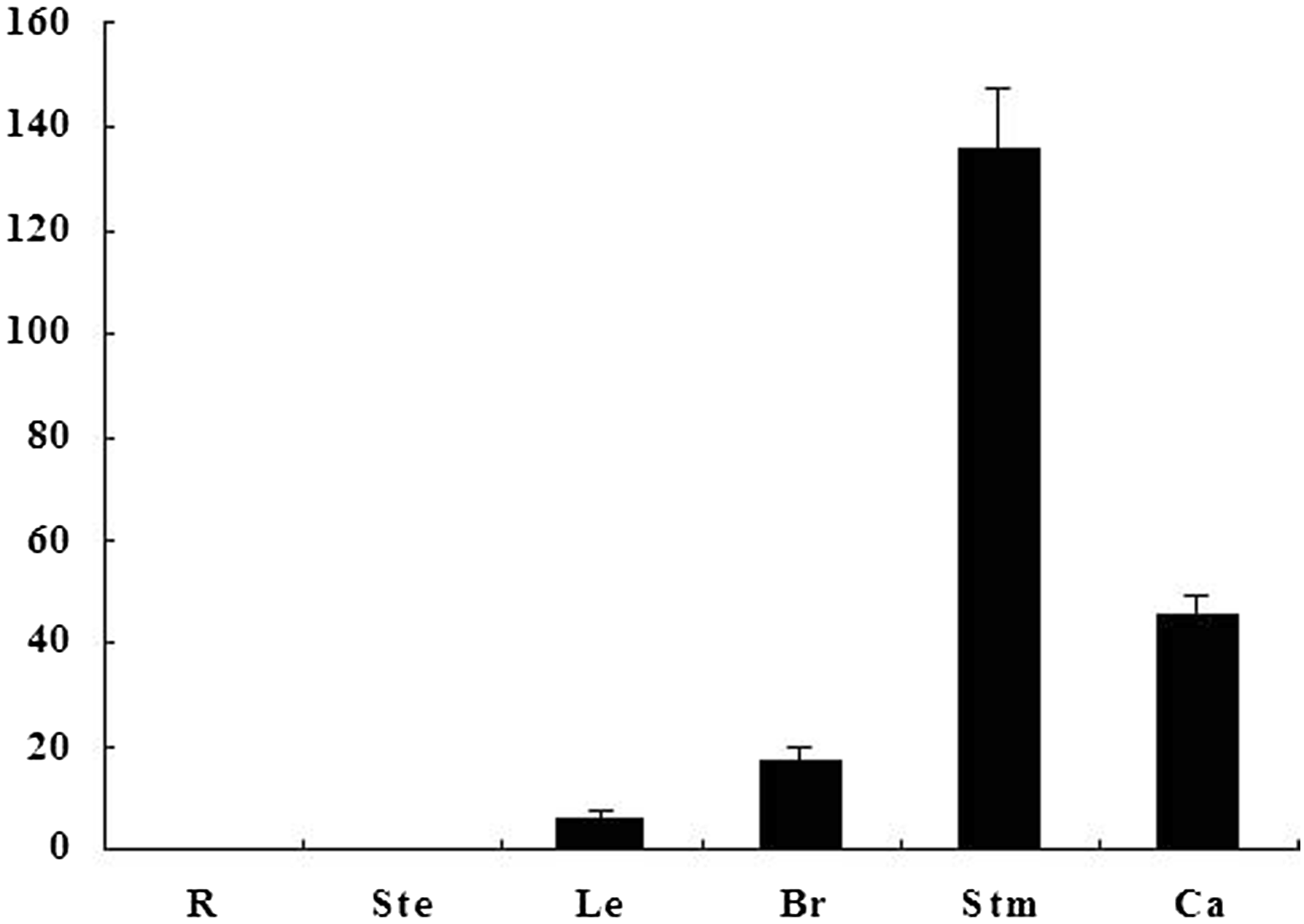

Supplement: Supplementary file 1 — Authors’ original file for figure 1 [file 40529_2013_70_MOESM1_ESM.tif]

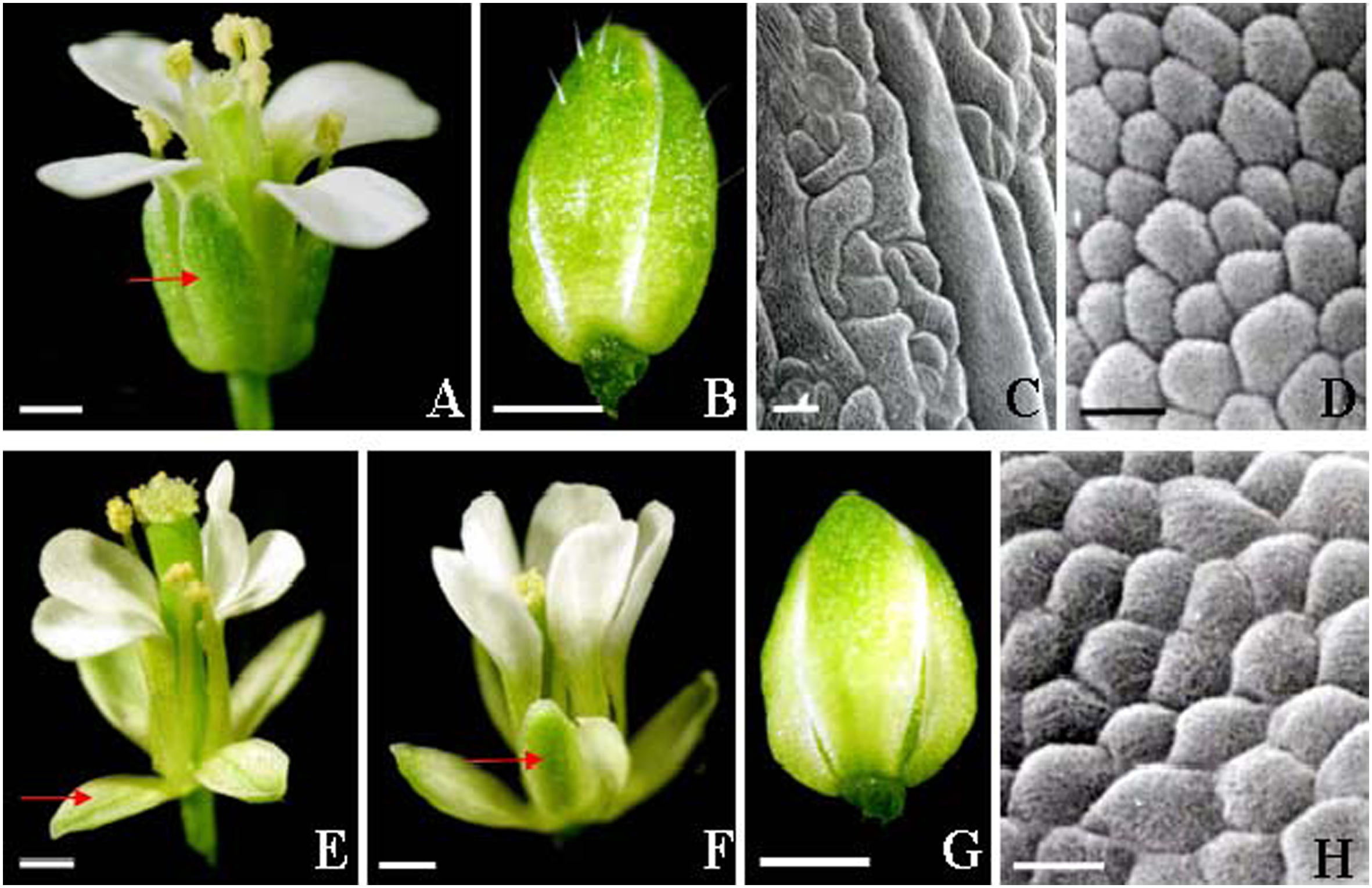

Supplement: Supplementary file 2 — Authors’ original file for figure 2 [file 40529_2013_70_MOESM2_ESM.tif]

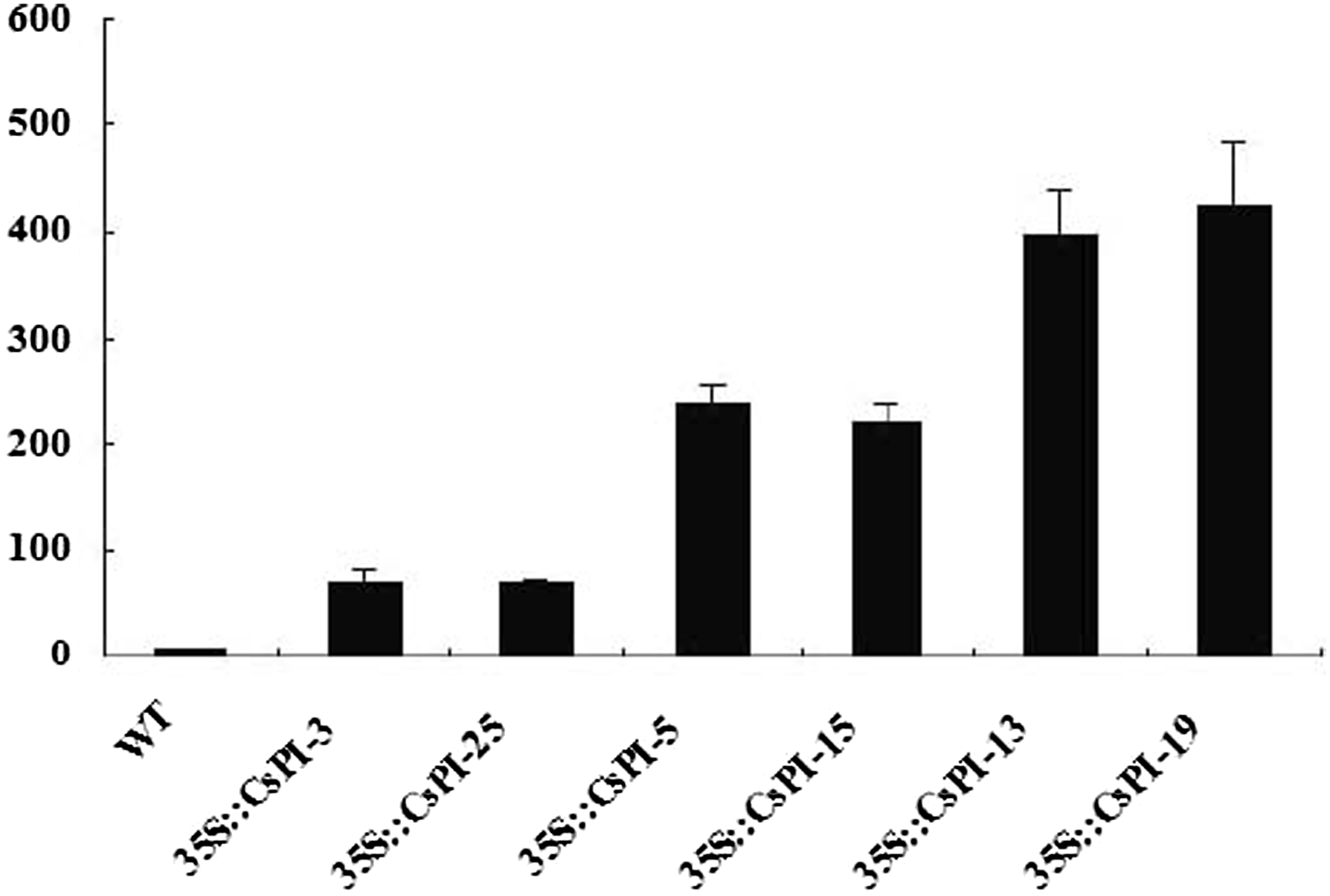

Supplement: Supplementary file 3 — Authors’ original file for figure 3 [file 40529_2013_70_MOESM3_ESM.tif]

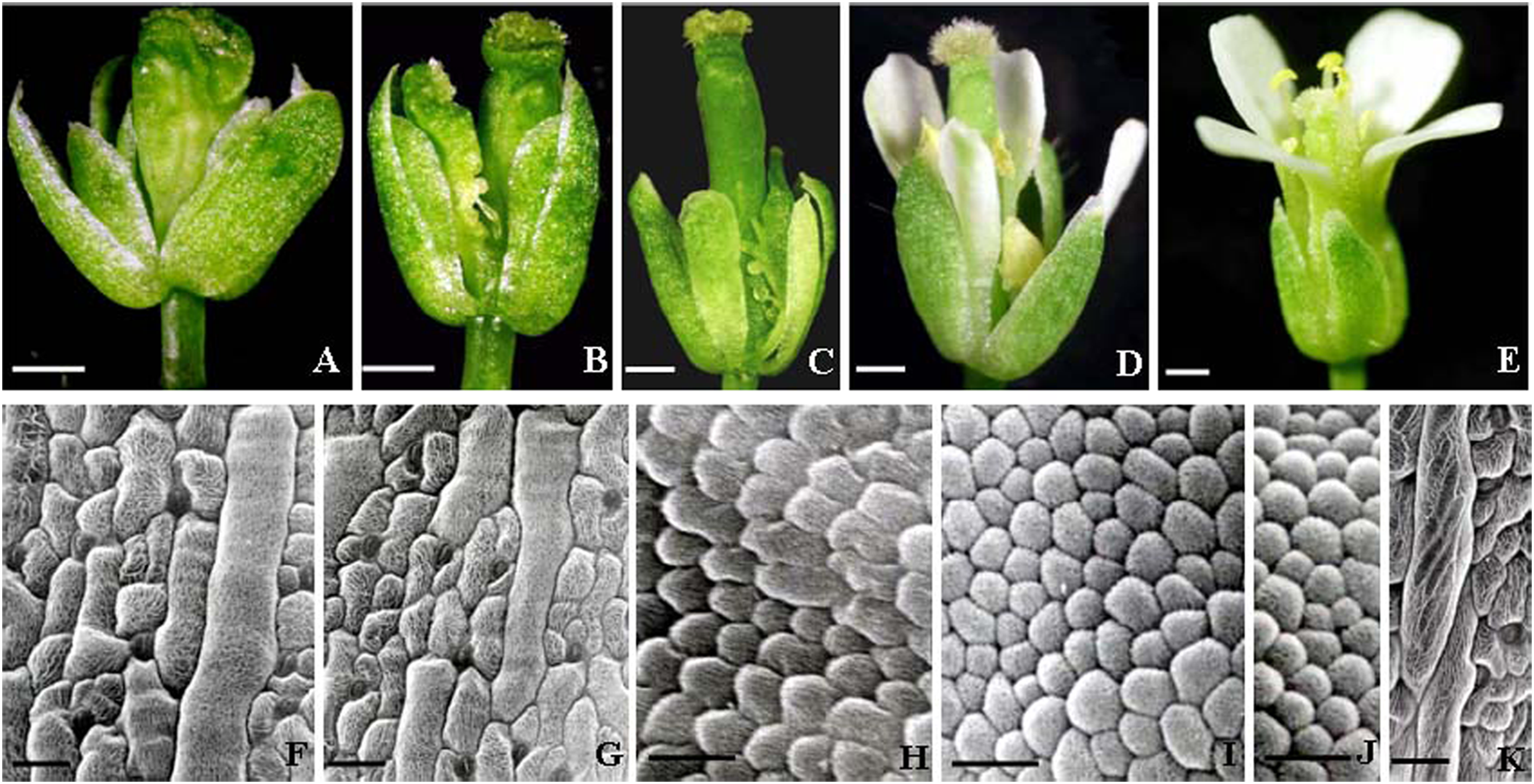

Supplement: Supplementary file 4 — Authors’ original file for figure 4 [file 40529_2013_70_MOESM4_ESM.tif]

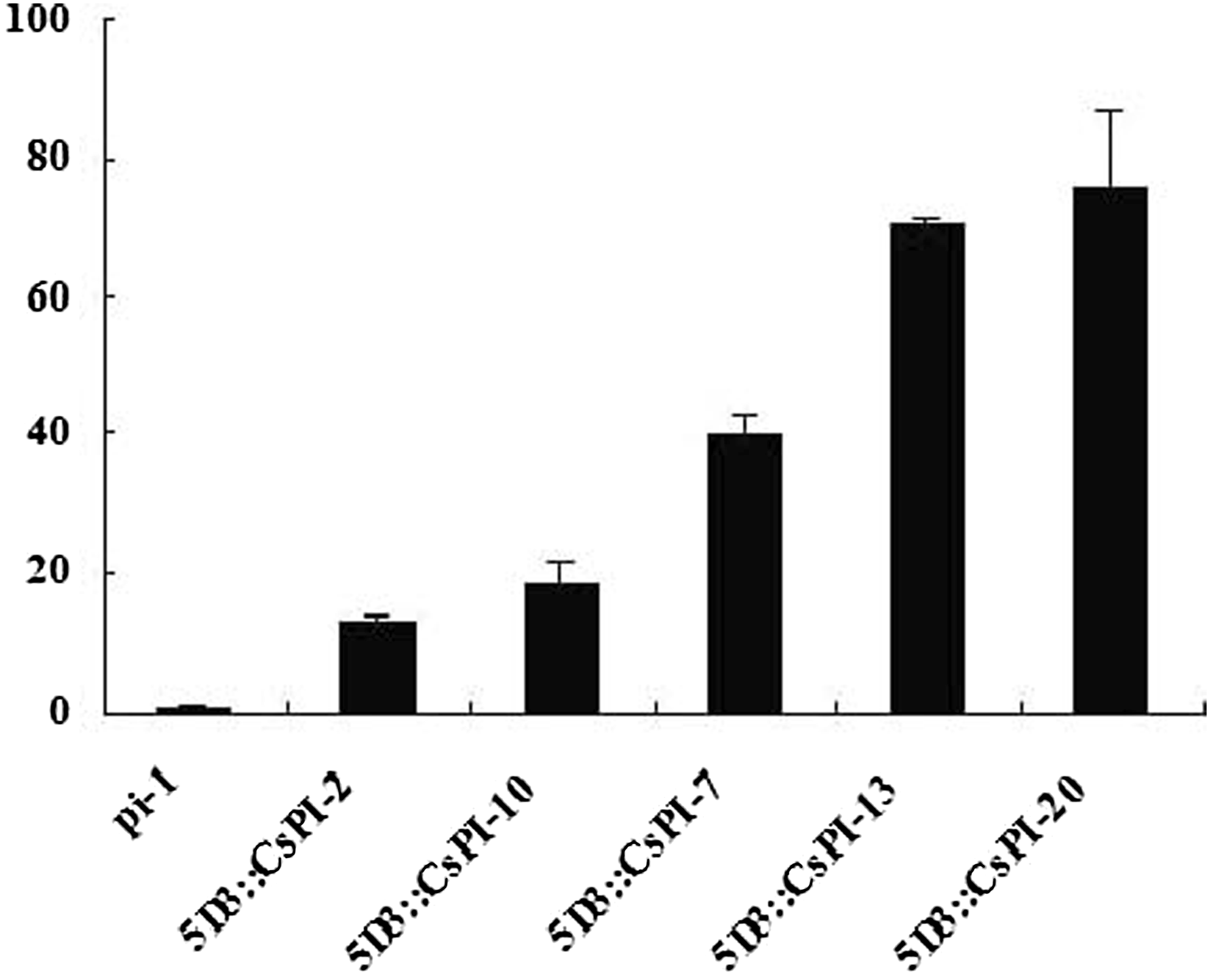

Supplement: Supplementary file 5 — Authors’ original file for figure 5 [file 40529_2013_70_MOESM5_ESM.tif]

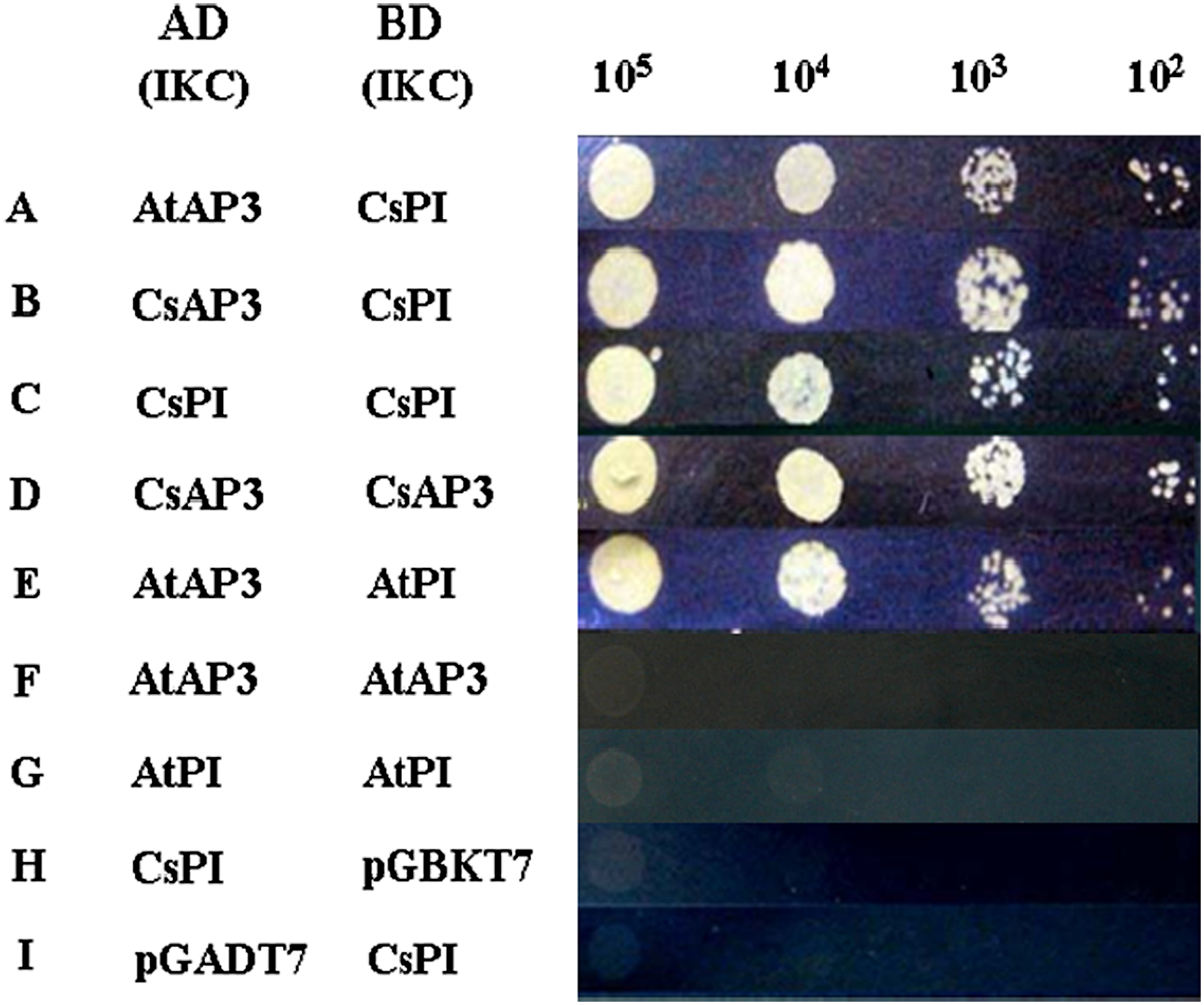

Supplement: Supplementary file 6 — Authors’ original file for figure 6 [file 40529_2013_70_MOESM6_ESM.tif]
